# Integrative Analyses Identify Potential Key Genes and Calcium-Signaling Pathway in Familial Atrioventricular Nodal Reentrant Tachycardia Using Whole-Exome Sequencing

**DOI:** 10.3389/fcvm.2022.910826

**Published:** 2022-07-18

**Authors:** Jichang Huang, Rong Luo, Chenqing Zheng, Xin Cao, Yuncai Zhu, Tao He, Mingjiang Liu, Zhenglin Yang, Xiushan Wu, Xiaoping Li

**Affiliations:** ^1^Institute of Geriatric Cardiovascular Disease, Chengdu Medical College, Chengdu, China; ^2^State Key Laboratory of Biocontrol, School of Life Sciences, Sun Yat-sen University, Guangzhou, China; ^3^School of Acupuncture-Moxibustion and Tuina, Chengdu University of Traditional Chinese Medicine, Chengdu, China; ^4^Department of Cardiology, Sichuan Academy of Medical Sciences and Sichuan Provincial People’s Hospital, University of Electronic Science and Technology of China, Chengdu, China; ^5^The Sichuan Provincial Key Laboratory of Human Disease Study, Institute of Laboratory Medicine, Sichuan Provincial People’s Hospital, University of Electronic Science and Technology of China, Chengdu, China; ^6^The Center for Heart Development, Hunan Normal University, Changsha, China; ^7^Guangdong Provincial Key Laboratory of Pathogenesis, Targeted Prevention and Treatment of Heart Disease, Guangzhou, China

**Keywords:** familial AVNRT, arrhythmia, whole-exome sequencing, pathogenic genes, calcium-signaling pathway

## Abstract

**Background:**

Atrioventricular nodal reentrant tachycardia (AVNRT) is a common arrhythmia. Growing evidence suggests that family aggregation and genetic factors are involved in AVNRT. However, in families with a history of AVNRT, disease-causing genes have not been reported.

**Objective:**

To investigate the genetic contribution of familial AVNRT using a whole-exome sequencing (WES) approach.

**Methods:**

Blood samples were collected from 20 patients from nine families with a history of AVNRT and 100 control participants, and we systematically analyzed mutation profiles using WES. Gene-based burden analysis, integration of previous sporadic AVNRT data, pedigree-based co-segregation, protein-protein interaction network analysis, single-cell RNA sequencing, and confirmation of animal phenotype were performed.

**Results:**

Among 95 related reference genes, seven candidate pathogenic genes have been identified both in sporadic and familial AVNRT, including *CASQ2*, *AGXT*, *ANK2*, *SYNE2*, *ZFHX3*, *GJD3*, and *SCN4A*. Among the 37 reference genes from sporadic AVNRT, five candidate pathogenic genes were identified in patients with both familial and sporadic AVNRT: *LAMC1*, ryanodine receptor 2 (*RYR2*), *COL4A3*, *NOS1*, and *ATP2C2*. To identify the common pathogenic mechanisms in all AVNRT cases, five pathogenic genes were identified in patients with both familial and sporadic AVNRT: *LAMC1*, *RYR2*, *COL4A3*, *NOS1*, and *ATP2C2*. Considering the unique internal candidate pathogenic gene within pedigrees, three genes, *TRDN*, *CASQ2*, and *WNK1*, were likely to be the pathogenic genes in familial AVNRT. Notably, the core calcium-signaling pathway may be closely associated with the occurrence of AVNRT, including *CASQ2*, *RYR2*, *TRDN, NOS1*, *ANK2*, and *ATP2C2*.

**Conclusion:**

Our pedigree-based studies demonstrate that *RYR2* and related calcium signaling pathway play a critical role in the pathogenesis of familial AVNRT using the WES approach.

## Introduction

Atrioventricular nodal reentrant tachycardia (AVNRT) is a relatively common arrhythmia, accounting for approximately 45–65% of paroxysmal supraventricular tachycardia (PSVT; 1). The heart rate of a normal adult is typically 60 to 100 beats per minute, whereas the heart rate of patients with AVNRT exceeds 150 beats per minute (2–4). This continuous re-excitement of the myocardium can induce arrhythmias, syncope, and even sudden death.

Slow and fast atrioventricular nodal pathways are currently recognized as the pathobiological mechanism for AVNRT, wherein the calcium-signaling pathway may be a crucial regulator (5). Calmodulin-dependent protein kinase II (CaMKII) can directly phosphorylate L-type voltage-gated calcium channels (Cav1.2) to increase Ca^2+^ influx in cardiomyocytes (6), inducing early depolarization and causing arrhythmia (7). In addition, CaMKII can phosphorylate the ryanodine receptor 2 (*RYR2*) on the sarcoplasmic reticulum (SR) to release a large amount of Ca^2+^ into the cytoplasm from SR (8). Excessive Ca^2+^ activates the Na^+^/Ca^2+^ exchanger (NCX), resulting in spontaneous myocyte depolarization and abnormal rhythm (8). Furthermore, the inhibition of NO synthase 1 (NOS1) in SR decreased *RYR2* activity because of reducing Ca^2+^ sparks and shortened action potential causing arrhythmia susceptibility (9). Although radiofrequency ablation for the treatment of AVNRT has shown good results, its precise reentry path and its molecular mechanism remain to be explained.

Atrioventricular nodal reentrant tachycardia was considered a sporadic disease in the past, with a prevalence of 22.5 cases per 10,000 persons (10). Nevertheless, several studies have reported that AVNRT occurred in twins and the same family member (10–13), indicating the phenomenon of family clustering of AVNRT. To date, few studies of AVNRT pedigrees have been available (10, 12, 14, 15), as this is relatively a rare phenomenon. Familial AVNRT pedigree was reported for the first time in 2004 (12). Subsequently, the European clinical study reported 24 AVNRT pedigrees in 2017 (10). Recently, we described the clinical reports of eight families with a history of AVNRT in China in 2021 (15).

The familial AVNRT phenomenon indicates that genetic factors play a crucial role in AVNRT pathogenesis; however, investigations at a molecular level are currently lacking. No report is available on the pathogenic genes of AVNRT. In addition, only two studies have explored the screening of pathogenic genes of AVNRT (5, 16). In 2018, Andreasen et al. first sequenced 67 known pathogenic genes associated with arrhythmia in 298 patients with AVNRT and reported mutations in genes encoding various Na^+^ and Ca^2+^ channels (16), suggesting that AVNRT is associated with various ion channels. Recently, we found that AVNRT is closely associated with the neuronal system or ion channels, and 10 potential candidate pathogenic genes were screened out in 82 patients with sporadic AVNRT using whole-exome sequencing (WES; 5). Although variants of genes were identified in patients with sporadic AVNRT, it is difficult to identify the disease phenotype and genotype. Fortunately, the emergence of pedigree-based studies addressed this issue (17). The pedigree-based study had several advantages for a rare variant: reduced genetic heterogeneity, enriched rare alleles, and co-segregated with the disease phenotype and genotype (18). Therefore, we hypothesized that the application of a more integrated approach might help elucidate the genetic etiology of AVNRT disease.

To the best of our knowledge, this is the first study that primarily aimed to investigate the genetic contribution of familial AVNRT using a WES approach. In this study, we used WES to identify potential key genes on the basis of gene-based burden, pedigree-based co-segregation, protein-protein interaction (PPI) analyses, single-cell RNA sequencing, and confirmation of phenotype for AVNRT disease.

## Materials and Methods

### Collection of Peripheral Blood Samples

Patients with AVNRT were enrolled in the Sichuan Provincial People’s Hospital in China from 2013 to 2020. Familial AVNRT defined that two or more AVNRT patients in a family, or 1 or more clinically diagnosed PSVT patients in a family of AVNRT proband patient. In addition, 100 unrelated ethnically matched healthy participants were recruited from the Sichuan Provincial People’s Hospital. All probands were diagnosed with AVNRT using intracardiac electrophysiological examination during treatment with radiofrequency catheter ablation, and family members diagnosed with AVNRT underwent intracardiac electrophysiological or transesophageal atrial pacing examination. Healthy participants did not have a history of cardiovascular diseases, arrhythmia, systemic immune diseases, cancers, or any other diseases known to cause arrhythmias. Whole blood samples from 20 patients with AVNRT and 100 normal control participants were collected in heparinized vacutainer tubes. Patients had signed an informed consent form before enrollment. This study was approved by the ethics committee of the Sichuan Academy of Medical Sciences and the Sichuan Provincial People’s Hospital.

### Intracardiac Electrophysiological Study

Intracardiac electrophysiology recordings included atrial stimulation (burst or additional stimulation pacing) and ventricular stimulation in patients. AVNRT diagnosis is established on the basis of published standards and applicable pacing operations. The physiology of dual atrioventricular node is defined as the atrial-His (AH) interval increase of ≥50 ms after a decreasing interval of 10 ms during the additional stimulation of the single atrium or the AH interval increase of ≥50 ms after the pacing cycle length is shortened by 10 ms. If continuous AVNRT is not induced (lasting more than 30 s), the same pacing procedure was repeated with isoproterenol administration as described above.

### Whole-Exome Sequencing, Variant Selection, and Annotation

In brief, we purified DNA from the peripheral blood using the QIAamp DNA Blood Mini Kit (Qiagen, Hilden, Germany). Whole-exome enrichment was performed using the SureSelect Human All Exon kit V6 (Agilent Technologies, Santa Clara, CA, United States). The genomic DNA library was sequenced using the HiSeq X and NovaSeq systems (Illumina, San Diego, CA, United States).

The sequenced DNA fragments were aligned with Human Reference Genome (National Center for Biotechnology Information Build 37) on the basis of the Burrows–Wheeler transform. The removal of duplication, realignment, and recalibration were performed with Picard tools^1^ and GATK^2^.

The single-nucleotide polymorphisms and insertion-deletion polymorphisms (indels) were performed using GATK3.7 software. The high-confidence variants were annotated with snpEff (Version 4.2)^3^. In addition, the annotations of all variants were further performed using 1000 Genomes Project data (2014 Oct release)^4^, the Exome Aggregation Consortium^5^, EVS^6^, the ClinVar^7^ database, and Online Mendelian Inheritance in Man^8^.

### Rare Variants of the Pathogenic Reference Genes

In total, 95 related reference genes as arrhythmia were selected for the analysis of rare variants in patients with AVNRT and control participants (5). These genes were considered reference genes according to our previous study (5). To increase reliability and generalizability of reference genes, data integration was used to select the genes following our previous sporadic AVNRT study. Therefore, the reference genes were identified in patients with both the sporadic and familial AVNRT and were assessed for segregation within families.

Biological process (BP) of Gene Ontology (GO) was performed by database for annotation, visualization and integrated discovery (DAVID) bioinformatics resources according to previous study (19). PPI network of candidate genes were obtained from the STRING database^9^. The images of single-cell sequencing data from healthy human cardiac tissue were obtained from the Human Protein Atlas^10^. The mouse phenotypes associated with pathogenic reference genes were extracted from the Mouse Genome Informatics (MGI) database^11^.

### Rare Variants of Common Pathogenic Genes in Sporadic and Familial Atrioventricular Nodal Reentrant Tachycardia

To identify the common pathogenic mechanisms in all AVNRT cases, the 37 most likely pathogenic genes from our previously sporadic AVNRT study (5) were considered the intersection of both sporadic and familial AVNRT, and then candidate pathogenic genes were identified in patients with both familial and sporadic AVNRT, and were assessed for segregation within families.

Biological process analysis was performed by DAVID bioinformatics resources (19). The PPI network of candidate genes was obtained from the STRING database (see text footnote 9). The images of single-cell sequencing data from 24 healthy human tissues are obtained from the Human Protein Atlas (see text footnote 10). There are 51 cell types of human tissues. The mouse phenotypes associated with pathogenic reference genes were extracted from the MGI database (see text footnote 11).

### Rare Variants of Potential Pathogenic Genes in Familial Atrioventricular Nodal Reentrant Tachycardia

To analyze the aggregate association of rare variants at the gene level, we performed gene-based burden analysis to obtain gene-level significant associations of familial AVNRT patients (*n* = 20) and control subjects (*n* = 100). Rare variants were defined as “deleterious variants” according to 1000 Genomes Project data and ExAC with MAF < 0.001, MAF < 0.01, or MAF < 0.05. Fisher’s exact test was used to evaluate gene-based burden analysis. The gene level across the genome was used to identify risk genes across different allele frequency spectrums.

The significant genes were submitted to the KOBAS3.0 web server^12^ to obtain the functional gene set Reactome Pathway enrichment. The PPI network of candidate genes was obtained from the STRING database (see text footnote 9). The images of single-cell sequencing data from healthy human cardiac tissue were obtained from the Human Protein Atlas (see text footnote 10). The mouse phenotypes associated with pathogenic reference genes were extracted from the MGI database (see text footnote 11).

### Protein–Protein Interactions Network of Potential Pathogenic Genes

Protein–protein interactions network of candidate genes were obtained from the STRING database (see text footnote 9). The relationships among the screened genes were predicted by STRING database and visualized with Cytoscape v2.3 software.

## Results

### Clinical Data of the Patients

**FIGURE 1:**
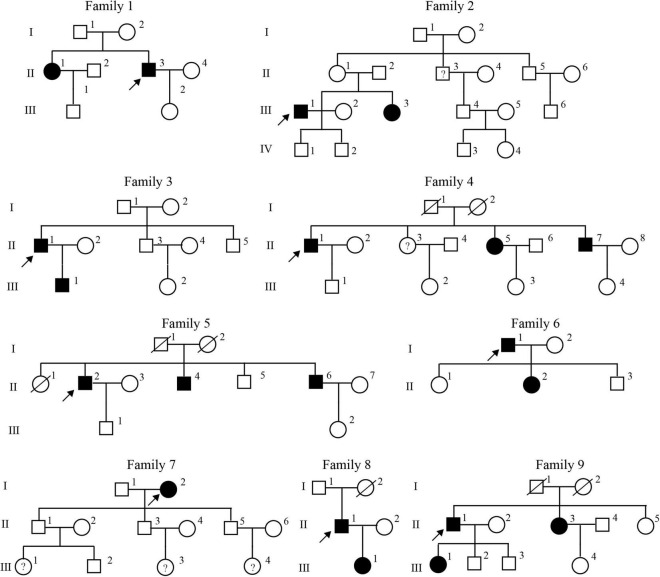
Pedigrees of the nine families with familial atrioventricular nodal reentrant tachycardia. The open squares and circles indicate the normal male and female members, respectively. The filled squares and circles indicate the affected male and female members, respectively. Arrow denotes a proband, and WES investigated the affected individuals. Question mark (?) denotes clinically diagnosed PSVT.

In this study, a total of 20 patients and 100 control participants were included to perform WES. These 20 patients were assessed in nine families, including a total of 93 members (Figure 1). Among 20 patients enrolled in this study, the male to female ratio was 1.86, the mean age at onset was approximately 47.5 years, the heart rate at onset was approximately 176.9 beats per minute, and all the patients were free from structural heart disease (Table 1). All the patients showed typical slow-fast AVNRT, and 60.0% of the patients were successfully treated by radiofrequency ablation during the operation.

**TABLE 1:** Demographic baseline of patients.

Variables	Total patients (*n* = 20)
Sex, male (%)	12 (60.0)
Age at onset, year	47.5 ± 14.3
Heart rate at onset, bpm	176.9 ± 12.8
Structural heart disease, yes (%)	0 (0)
AVNRT Type, typical (%)	20 (100)
Radiofrequency ablation, yes (%)	12 (60.0)

*bpm: beat per minute.*

### Rare Variants of the Pathogenic Reference Genes

To increase reliability and generalizability of related reference genes, data integration was used to confirm the genes following our previous sporadic AVNRT study (5). Therefore, patients with both sporadic and familial AVNRT were enrolled in this study. Among the 95 related reference genes, seven candidate pathogenic genes have been identified in patients with both familial and sporadic AVNRT: *CASQ2*, *AGXT*, *ANK2*, *SYNE2*, *ZFHX3*, *GJD3*, and *SCN4A* (Supplementary Table 1). We found *CASQ2* have distinct feature between familial and sporadic AVNRT (1 rare variant, 6/20 patients, familial AVNRT; 1 rare variant, 1/82 patient, sporadic AVNRT). Furthermore, rare variants of *AGXT*, *ANK2*, *SYNE2*, *GJD3*, and *SCN4A* co-segregated within one pedigree and those of *CASQ2* and *ZFHX3* within two and three pedigrees, respectively (Supplementary Table 1).

**FIGURE 2:**
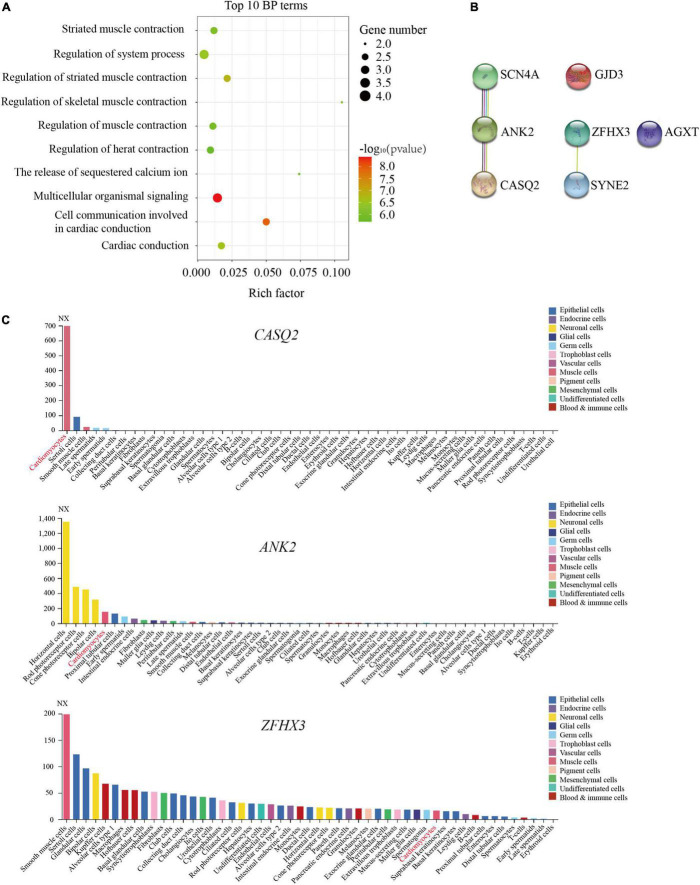
Identification of pathogenic reference genes in familial atrioventricular nodal reentrant tachycardia. **(A)** The top 10 biological process terms of seven pathogenic genes are depicted using enrichment analysis (*P* < 0.05). **(B)** The protein–protein interactions analysis of seven pathogenic genes. **(C)** The expression of *CASQ2*, *ANK2*, and *ZFHX3* was shown in different cell types by the single-cell sequencing data.

The bubble plot of GO-BP analysis showed that the functions of these genes were mainly associated with cardiac conduction, muscle contraction, and the release of sequestered calcium ions (Supplementary Table 1 and Figure 2A). Furthermore, PPI networks of these genes indicated that *CASQ2*, *ANK2*, and *SCN4A* constituted the network, and *ZFHX3* interacted with *SYNE2* (Figure 2B). In addition, the results of single-cell sequencing showed that the relative expression of *CASQ2*, *ANK2*, and *SYNE2* was higher in cardiomyocytes than others (Figure 2C and Supplementary Figure 1), whereas the expression of *SCN4A*, *ZFHX3*, *AGXT*, and *GJD3* was relatively lower or not expressed (Figure 2C and Supplementary Figure 1).

To further verify these gene functions, the MGI database was used to confirm their phenotype. The disruption of *CASQ2*, essential for Ca^2+^ storage, led to ventricular tachycardia in both mice and humans (Table 2). Moreover, the abnormal function of ankyrin-2 (*ANK2*) may lead to sinoatrial node disease and ankyrin-B-related cardiac arrhythmia in humans (Table 2). In addition, its abnormality increased heart rate variability and caused the abnormal sinoatrial node conduction in the mouse (Table 2). *ZFHX3* was identified as a crucial risk factor for atrial fibrillation (20), *SYNE2* contributed to cardiac arrhythmia (Table 2), and *GJD3* caused abnormal atrioventricular node conduction (Table 2).

**TABLE 2:** Phenotype of candidate pathogenic genes in MGI database.

Gene	Human phenotypes	Mouse genotype
*CASQ2*	Ventricular tachycardia	Ventricular tachycardia
	Syncope	Abnormal sinus arrhythmia
	Bradycardia	Abnormal cardiac muscle relaxation
*ANK2*	Atrial fibrillation	Increased heart rate variability
	Sudden cardiac death	Abnormal sinoatrial node conduction
	Sinus bradycardia	None
	Syncope	None
*SYNE2*	Arrhythmia	Abnormal retinal blood vessel morphology
	Cardiomyopathy	None
*GJD3*	None	Abnormal impulse conducting system conduction
	None	Abnormal atrioventricular node conduction
	None	Shortened PQ interval
*NOS1*	None	Increased heart rate
	None	Acardiac muscle relaxation
*RYR2*	Ventricular arrhythmia	Ventricular tachycardia
	Ventricular tachycardia	Increased heart rate
*COL4A3*	Hypertension	Abnormal glomerular capillary morphology
*LAMC1*	None	Intracranial hemorrhage
*TRDN*	Ventricular tachycardia	None
*ANO6*	None	Shortened PQ interval

Based on MGI database and previous study (20), we suggested that *CASQ2, ANK2*, *SYNE2*, *GJD3*, and *ZFHX3* were the most likely pathogenic genes for AVNRT.

### Rare Variants of Common Pathogenic Genes in Sporadic and Familial Atrioventricular Nodal Reentrant Tachycardia

To identify the common pathogenic mechanisms in all AVNRT cases, the 37 most likely pathogenic genes from our previous sporadic AVNRT study (5) were considered the intersection of both sporadic and familial AVNRT. Among these genes, five pathogenic genes were identified in patients with both familial and sporadic AVNRT: *LAMC1*, *RYR2*, *COL4A3*, *NOS1*, and *ATP2C2* (Supplementary Table 2). We identified only one gene *NOS1* that was totally shared the same two rare variants both in six familial (6/20 patients) and 33 sporadic (33/82 patients) AVNRT patients (Supplementary Table 2). Other genes have distinct features between familial and sporadic AVNRT: *LAMC1* (1 rare variant, 2/20 patients, familial AVNRT; 4 rare variants, 7/82 patients, sporadic AVNRT), *RYR2* (1 rare variant, 1/20 patient, familial AVNRT; 8 rare variants, 8/82 patients, sporadic AVNRT), *COL4A3* (1 rare variant, 2/20 patients, familial AVNRT; 5 rare variants, 5/82 patients, sporadic AVNRT); *ATP2C2* (2 rare variants, 2/20 patients, familial AVNRT; 5 rare variants, 5/82 patients, sporadic AVNRT; Supplementary Table 2). Furthermore, the rare variants of *LAMC1*, *COL4A3*, *NOS1*, and *ATP2C2* co-segregated within one pedigree apart from *RYR2* (Supplementary Table 2).

**FIGURE 3:**
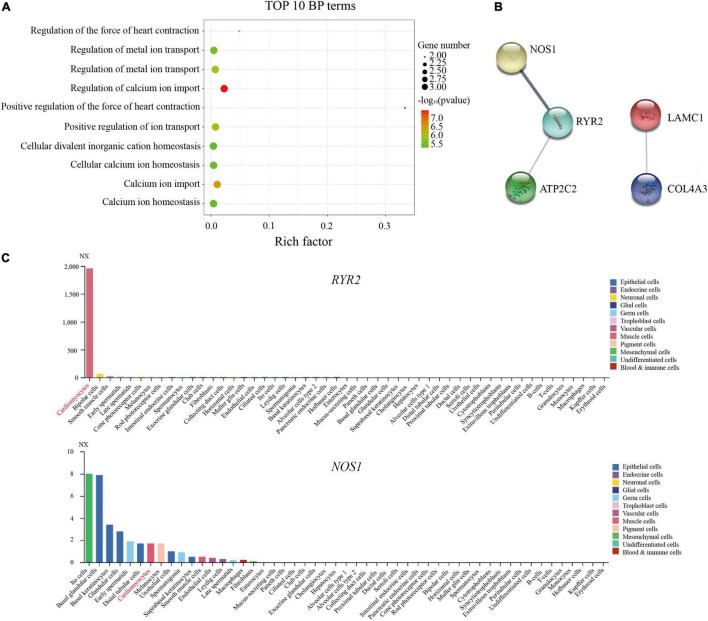
Identification of common pathogenic genes in sporadic and familial atrioventricular nodal reentrant tachycardia. **(A)** The top 10 biological process terms of five pathogenic genes are depicted using enrichment analysis (*P* < 0.05). **(B)** The protein–protein interactions analysis of five pathogenic genes. **(C)** The expression of *RYR2* and *NOS1* was shown in different cell types using the single-cell sequencing data.

The BP enrichment analysis suggested that the functions of these genes were mainly associated with heart contraction and the regulation of calcium ion (Figure 3A and Supplementary Table 2). In addition, the PPI networks showed that *RYR2*, *NOS1*, and *ATP2C2* constituted the network, and *COL4A3* interacted with *LAMC1* (Figure 3B). Moreover, the results of single-cell sequencing data showed that the relative expression of *RYR2* and *LAMC1* was higher in cardiomyocytes than others (Figure 3C and Supplementary Figure 2), whereas the expression of *COL4A3*, *NOS1*, and *ATP2C2* was relatively lower or not expressed (Figure 3C and Supplementary Figure 2).

The biological function and phenotype of these genes were further explored using the MGI database. Homozygous mutation in the *NOS1* gene led to abnormal cardiac muscle relaxation and increased heart rate in the mouse (Table 2). The disruption of *RYR2* was associated with ventricular dysplasia and ventricular tachycardia in humans, whereas it is mainly associated with an increased heart rate and ventricular tachycardia in the mouse (Table 2). However, cardiac diseases were independent of the functions of *LAMC1*, *COL4A3*, and *ATP2C2* (Table 2).

Considering their functions and previous study (5), *RYR2* and *NOS1* were likely to be causal genes for AVNRT.

### Rare Variants of Pathogenic Genes in Familial Atrioventricular Nodal Reentrant Tachycardia

In search of the underlying pathogenic mechanisms within AVNRT pedigrees, we imposed more restrictive criteria: more than two mutations and one homozygous mutation in one gene segregated at least two pedigrees. A total of 299 genes with 452 rare variants were identified (Supplementary Table 3).

As shown in Supplementary Table 3, the three AVNRT-related traits among the pathways in the Reactome databases were as follows: (1) stimuli-sensing channels, (2) RYR tetramers transport Ca^2+^ from the SR lumen to the cytosol, and (3) ion channel transport. In addition, seven pathogenic genes were identified, including *TRDN*, *ANO6*, *SLC9C1*, *CASQ2*, *ATP6V0A4*, *SGK2*, and *WNK1*. Remarkably, *CASQ2* has been involved in AVNRT as reference genes.

Mouse genome informatics database and previous studies was further used to confirm the phenotype of these genes. The disruption of *TRDN* contributed to ventricular tachycardia in humans (Table 2; 21). Mice lacking *ANO6* developed shortened PQ intervals (Table 2). The aberration of *WNK1* led to hereditary sensory and autonomic neuropathy in humans (22). Considering their functions and previous study (21, 22), *TRDN*, *CASQ2*, and *WNK1* were likely to be the common pathogenic genes in familial AVNRT.

### The Calcium-Signaling Pathway of Atrioventricular Nodal Reentrant Tachycardia

**FIGURE 4:**
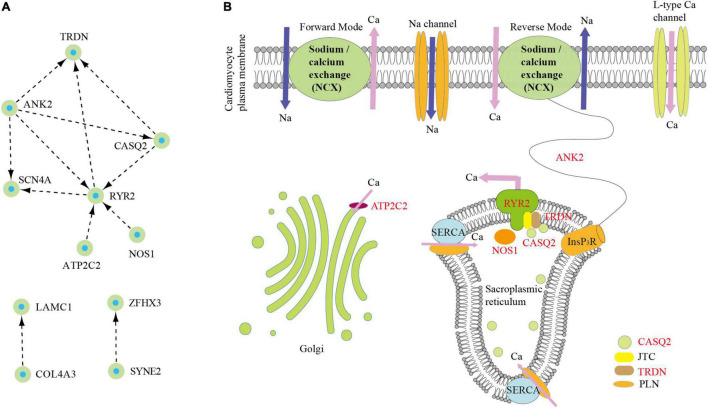
Identification of core-signaling pathway in atrioventricular nodal reentrant tachycardia (AVNRT). **(A)** The protein–protein interactions analysis of 14 pathogenic genes. **(B)** Schematic representation of core-signaling pathway in AVNRT.

To explore the internal relationship of the candidate pathogenic genes in this study, PPI network analysis was further constructed. Among these 14 candidate pathogenic genes, three networks were established; these genes were *CASQ2*, *AGXT*, *ANK2*, *SYNE2*, *ZFHX3*, *GJD3*, *SCN4A*, *LAMC1*, *RYR2*, *COL4A3*, *NOS1*, *ATP2C2, TRDN*, and *WNK1* (Figure 4A). PPI networks indicated that the genes constituted network 1 (*CASQ2*, *ANK2*, *SCN4A*, *RYR2*, *NOS1*, *ATP2C2*, and *TRDN*), network 2 (*SYNE2* and *ZFHX3*), and network 3 (*LAMC1* and *COL4A3*).

The maximum network 1 was mainly associated with the calcium-signaling pathway using Kyoto Encyclopedia of Genes and Genomes enrichment analysis (Figure 4B). Among them, *RYR2* acted as a calcium channel that released calcium ions into the cytoplasm from the SR and thus regulated cardiac muscle contraction (23). The RYR forms a complex with *TRDN*, junction (*JTC*), and *CASQ* instead of acting independently (24). Moreover, the mutations of *RYR2* or *CASQ* lead to Ca^2+^ leak in ventricular tachycardia and thus contribute to Ca^2+^ waves in arrhythmogenic as a result of the increasing Ca^2+^ spark frequency and rising flux (24, 25). Particularly, *CASQ2* and *TRDN* have also been identified in this study. Furthermore, another *RYR2*-related gene is neuronal *NOS1*, which is located in the cardiac SR and enhances contraction through NO production (26). The present studies have shown that the inhibition of *NOS1* decreased *RYR2* activity because of reducing Ca^2+^ sparks and shortened action potential causing arrhythmia susceptibility (9, 26). In addition, *ANK2* from the SR promotes the flow of calcium ions into the plasma membrane through the inositol triphosphate receptor and NCX (27). *ATP2C2* encodes calcium-transporting ATPase, removing calcium from the cytosol into the Golgi body (28). Therefore, the calcium-signaling pathway may be closely associated with the occurrence of AVNRT.

## Discussion

Although significant inroads have been achieved in elucidating the pathogenesis of AVNRT (1, 5), the molecular mechanisms associated with this disease remain in its early stages. The sporadic studies contributed to the discovery of a large number of candidate pathogenic genes (5); however, it is difficult to effectively rule out unrelated genes. Unlike the sporadic studies, the pedigree-based linkage study directly observes the segregation of variants with disease phenotype (17). The integrated analysis of sporadic and familiar studies may provide novel strategies for exploring the more prevalent pathogenesis. Phenotypes associated with pathogenic genes were further confirmed using the MGI database. Thus, we took advantage of phenotype analysis and integrated sporadic and pedigree analyses to reveal the novel genetic associations with AVNRT. In this study, genes such as *CASQ2, ANK2*, *ZFHX3*, *RYR2*, *NOS1*, *TRDN*, and *WNK1* were likely pathogenic.

Recently, accumulating studies have revealed that genetic factors may contribute to the pathogenesis of AVNRT (10–13). However, little is known about the genetic role of AVNRT. In 298 patients with AVNRT, the disease was observed to be associated with Na^+^ and Ca2^+^ channels detected using next-generation sequencing (16). Recently, we, for the first time, found that AVNRT was closely associated with the neuronal system or ion channels, and 10 potential candidate pathogenic genes were screened out in 82 patients with sporadic AVNRT using WES (5). Among the pathogenic reference genes, multiple variants in ion channel genes (*CASQ2*, *ANK2*, and *SCN4A*) were further confirmed both in previous sporadic (5) and these familial studies. The gene *CASQ2*, encoding the calcium-binding protein, played a crucial role in excitation-contraction coupling, regulated the heart rate, and was associated with ventricular tachycardia (29–31). Moreover, another calcium ion transport-related gene *ANK2* may lead to cardiac arrhythmia (32). *ZFHX3* was identified as a crucial risk factor for atrial fibrillation (33), and *SYNE2* contributed to atrial fibrillation (34). These results suggested calcium handling might have played a crucial role in the pathogenesis of AVNRT.

Slow and fast atrioventricular nodal pathways are currently recognized as the mainly pathobiological mechanism for AVNRT, wherein the calcium-signaling pathway may be a crucial regulator (5). CaMKII can directly phosphorylate L-type voltage-gated calcium channels (Cav1.2) to increase Ca^2+^ influx in cardiomyocytes, inducing early depolarization and causing an arrhythmia (6). Moreover, CaMKII can phosphorylate the *RYR2* on the SR to release a large amount of Ca^2+^ into the cytoplasm from the SR, and excessive Ca^2+^ activates the NCX, resulting in spontaneous myocyte depolarization and abnormal rhythm (8). Furthermore, the inhibition of *NOS1* in the SR decreased RYR2 activity because of reducing Ca^2+^ sparks and shortened action potential causing arrhythmia susceptibility (9).

In both sporadic and familial AVNRT, there were six calcium channel-associated genes, including *RYR2*, *NOS1*, *TRDN*, *CASQ2*, *ANK2*, and *ATP2C2*. *RYR2* acted as a calcium channel that released calcium ions into the cytoplasm from the SR and thus regulated cardiac muscle contraction (23). The RYR forms a complex with *TRDN*, *JTC*, and *CASQ* instead of acting independently (24). Moreover, the mutations of *RYR2* or *CASQ* lead to Ca^2+^ leak in ventricular tachycardia, thus contributing to Ca^2+^ waves in arrhythmogenic as a result of the increasing Ca^2+^ spark frequency and rising flux (24, 25). Particularly, *CASQ2* and *TRDN* have also been identified in this study. Furthermore, another *RYR2*-related gene was neuronal *NOS1*. *NOS1* is located in the cardiac SR and enhances contraction through NO production (26). The studies showed that the inhibition of *NOS1* decreased *RYR2* activity because of reducing Ca^2+^ sparks and shortened action potential causing arrhythmia susceptibility (9, 26). From these findings, *RYR2* as the core-signaling pathway may be closely associated with the occurrence of AVNRT. The functions of these calcium channel-associated genes are currently being explored in functional experiments.

To the best of our knowledge, this is the first study primarily aimed to investigate the genetic contribution of familial AVNRT using a WES approach. The calcium-signaling pathway should be considered seriously for AVNRT.

## Data Availability Statement

The data presented in this study are deposited in the CNGB Sequence Archive (CNSA) of China National GeneBank DataBase (CNGBdb) repository, accession number CNP0003176.

## Ethics Statement

This study was approved by the Ethics Committee of the Sichuan Academy of Medical Sciences and the Sichuan Provincial People’s Hospital. The patients/participants provided their written informed consent to participate in this study.

## Author Contributions

JH, RL, CZ, ZY, XW, and XL: conceptualization. JH, RL, and XL: methodology. JH and CZ: software. JH, RL, XC, and YZ: validation. YZ, TH, ML, and XL: investigation. JH and XL: writing—original draft preparation. JH, CZ, XW, and XL: writing—review and editing. ZY, XW, and XL: resources. XL: supervision and project administration. JH, RL, XW, and XL: funding acquisition. All authors have read and agreed to the published version of the manuscript.

## Conflict of Interest

The authors declare that the research was conducted in the absence of any commercial or financial relationships that could be construed as a potential conflict of interest.

## Publisher’s Note

All claims expressed in this article are solely those of the authors and do not necessarily represent those of their affiliated organizations, or those of the publisher, the editors and the reviewers. Any product that may be evaluated in this article, or claim that may be made by its manufacturer, is not guaranteed or endorsed by the publisher.

## Footnotes

1
http://picard.sourceforge.net/

2
http://www.broadinstitute.org/gsa/wiki/index.php/Home_Page

3
http://snpeff.sourceforge.net/

4
http://www.1000genomes.org

5
http://exac.broadinstitute.org

6
http://evs.gs.washington.edu/EVS

7
http://www.ncbi.nlm.nih.gov/clinvar

8
http://www.omim.org

9
https://cn.string-db.org

10
http://www.proteinatlas.org

11
http://www.informatics.jax.org/

12
http://kobas.cbi.pku.edu.cn/kobas3

## Supplementary Material

Supplementary Figure 1 The expression of pathogenic reference genes in familial AVNRT. The expression of *SCN4A*, *SYNE2*, *AGXT*, and GJD3 was shown in different cell types by the single-cell sequencing data.

Supplementary Figure 2 The expression of common pathogenic genes in sporadic and familial AVNRT. The expression of *LAMC1*, *COL4A3*, and *ATP2C2* was shown in different cell types by the single-cell sequencing data.
