# Supplementary figures and images for: Integrative Analyses Identify Potential Key Genes and Calcium-Signaling Pathway in Familial Atrioventricular Nodal Reentrant Tachycardia Using Whole-Exome Sequencing
Source: Front Cardiovasc Med. 2022 Jul 18;9:910826. doi: 10.3389/fcvm.2022.910826 (PMC9339905; doi:10.3389/fcvm.2022.910826)

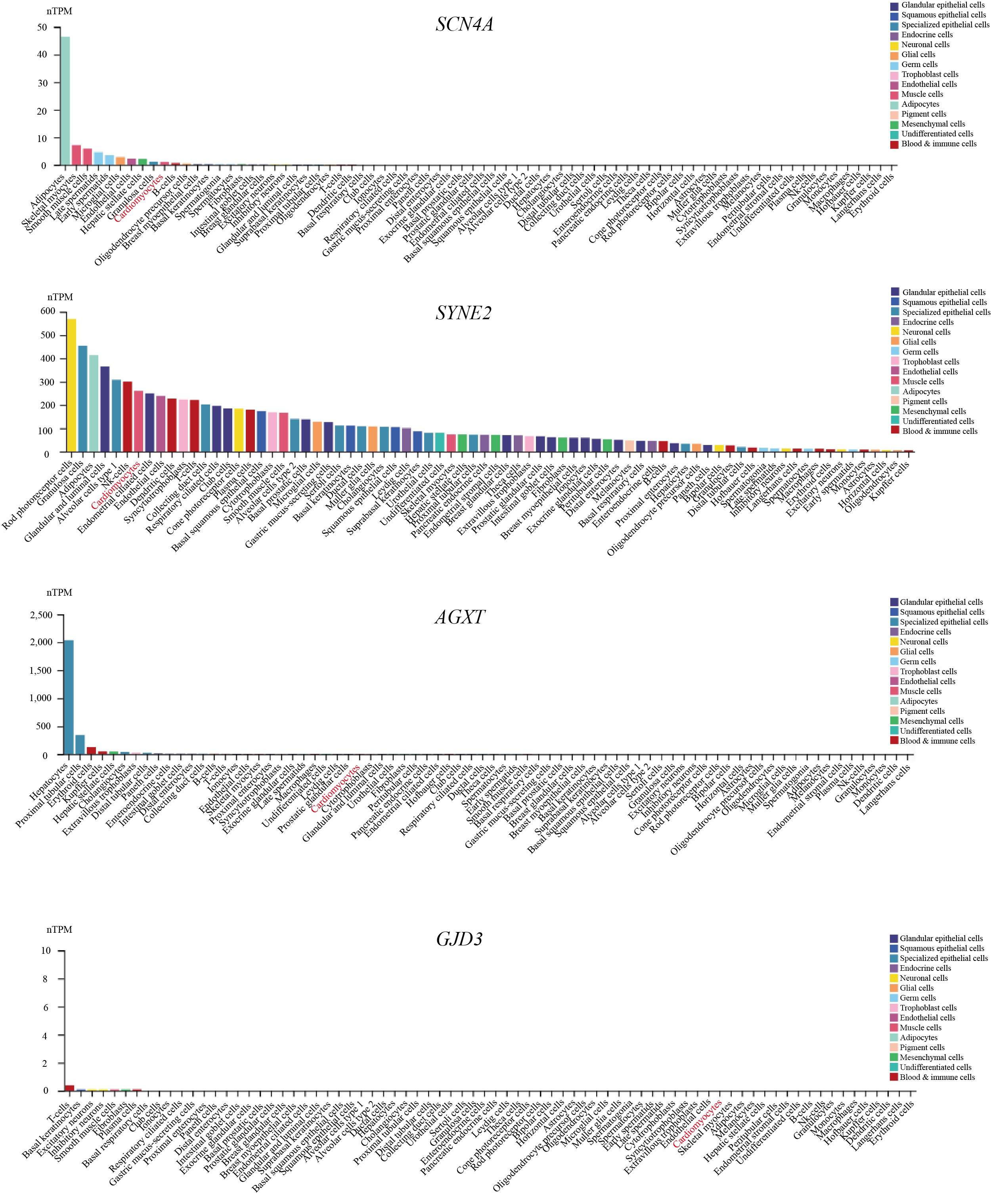

Supplement: Supplementary Figure 1 — The expression of pathogenic reference genes in familial AVNRT. The expression of SCN4A, SYNE2, AGXT, and GJD3 was shown in different cell types by the single-cell sequencing data. [file Image_1.JPEG]

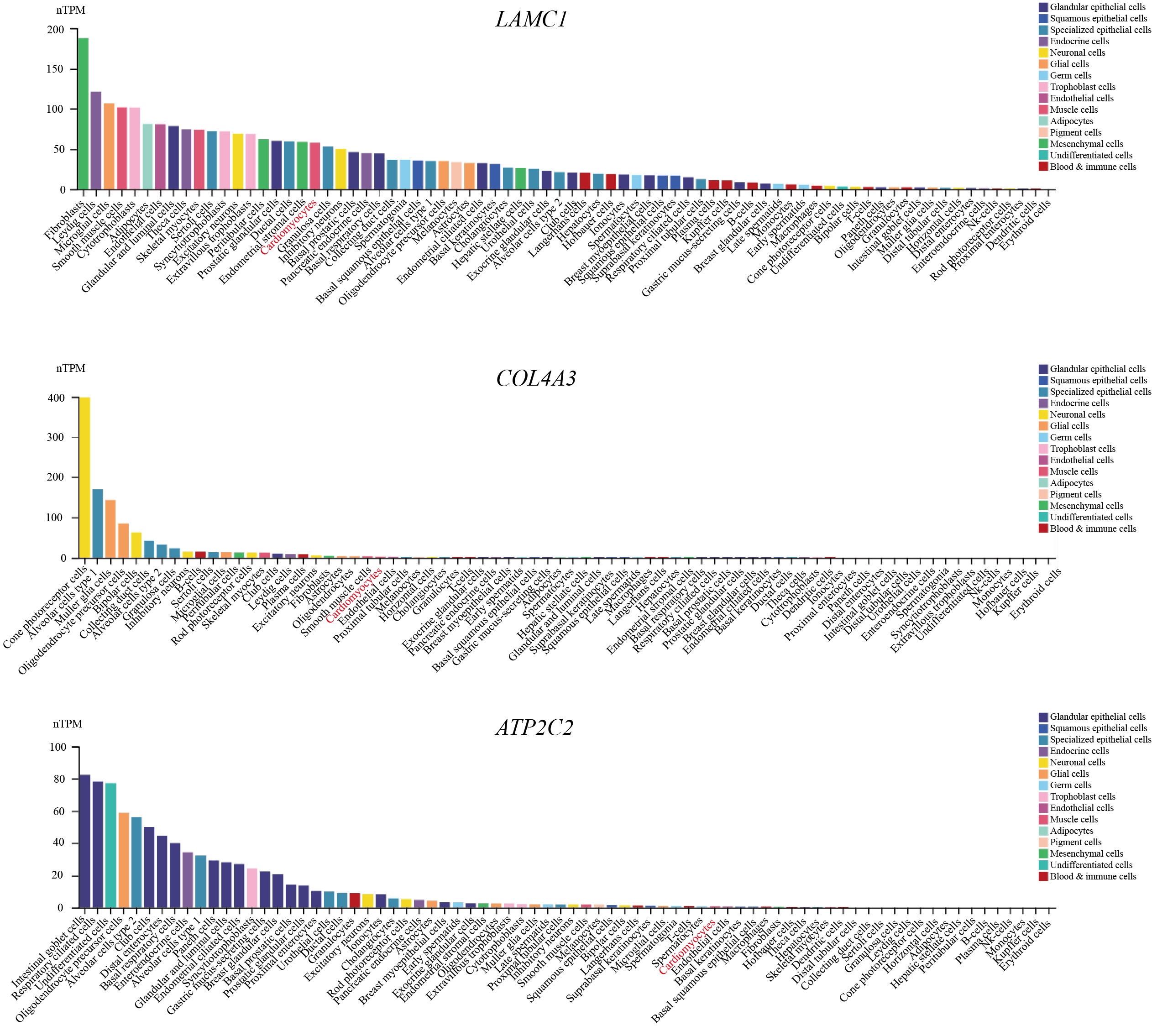

Supplement: Supplementary Figure 2 — The expression of common pathogenic genes in sporadic and familial AVNRT. The expression of LAMC1, COL4A3, and ATP2C2 was shown in different cell types by the single-cell sequencing data. [file Image_2.JPEG]
